# BHLHE22 Expression Is Associated with a Proinflammatory Immune Microenvironment and Confers a Favorable Prognosis in Endometrial Cancer

**DOI:** 10.3390/ijms23137158

**Published:** 2022-06-28

**Authors:** Darmawi, Lin-Yu Chen, Po-Hsuan Su, Phui-Ly Liew, Hui-Chen Wang, Yu-Chun Weng, Rui-Lan Huang, Hung-Cheng Lai

**Affiliations:** 1 International Ph.D. Program in Medicine, College of Medicine, Taipei Medical University, Taipei 110, Taiwan; d142108012@tmu.edu.tw; 2 Translational Epigenetics Center, Shuang Ho Hospital, Taipei Medical University, New Taipei 235, Taiwan; maple916chen@gmail.com (L.-Y.C.); pohsuansu@gmail.com (P.-H.S.); kxther@gmail.com (Y.-C.W.); gyntsgh@gmail.com (R.-L.H.); 3 Department of Histology, Faculty of Medicine, Universitas Riau, Pekanbaru 28293, Indonesia; 4 Department of Obstetrics and Gynecology, Shuang Ho Hospital, Taipei Medical University, New Taipei 235, Taiwan; 5 Department of Pathology, Shuang Ho Hospital, Taipei Medical University, New Taipei 235, Taiwan; lilyliew@tmu.edu.tw; 6 Department of Pathology, School of Medicine, College of Medicine, Taipei Medical University, Taipei 110, Taiwan; 7 Department of Obstetrics and Gynecology, College of Medicine, Taipei Medical University, Taipei 110, Taiwan; egg-0420@yahoo.com.tw; 8 Department of Obstetrics and Gynecology, School of Medicine, College of Medicine, National Defense Medical Center, Taipei 114, Taiwan

**Keywords:** BHLHE22, endometrial cancer, prognostic, immune infiltrating cells

## Abstract

Endometrial cancer (EC) rates are rising annually. Additional prediction markers need to be evaluated because only 10–20% of EC cases show an objective response to immune-checkpoint inhibitors (ICIs). Our previous methylomic study found that BHLHE22 is hypermethylated in EC tissues and can be detected using a Pap-smear sample. BHLHE22, a basic helix loop helix transcription factor family member, is known as a transcriptional repressor and is involved in cell differentiation. However, the role of BHLHE22 in EC remains poorly understood. Herein, we analyzed BHLHE22 expression in 54 paired cancer and normal endometrial tissue samples, and confirmed with databases (TCGA, GTEx, and human protein atlas). We found that BHLHE22 protein expression was significantly downregulated in EC compared with normal endometrium. High BHLHE22 expression was associated with microsatellite-instable subtype, endometrioid type, grade, and age. It showed a significant favorable survival. BHLHE22 overexpression inhibited the proliferation and migration of EC cells. Functional enrichment analysis showed that BHLHE22 was significantly associated with immune-related pathways. Furthermore, BHLHE22 was positively correlated with proinflammatory leukocyte infiltration and expression of chemokine genes in EC. In conclusion, BHLHE22 regulates immune-related pathways and modulates the immune microenvironment of EC.

## 1. Introduction

Endometrial cancer (EC) is the second most common and third most deadly malignancy among gynecological cancers [1]. In 2021, the American Cancer Society estimated that there were 66,570 new cases and 12,940 deaths in contrast to 2015, when there were an estimated 54,870 new cases and 10,170 deaths [2,3]. The rise in obesity and decline in hysterectomies are the most potent factors influencing these trends in EC [4,5]. Based on data from the US Surveillance, Epidemiology, and End Results (SEER) database from 2011 to 2017, the five-year overall survival rate for EC are 94.9%, 69.3%, and 17.8% for local, regional, and distant EC rates, respectively [6]. Advanced-stage and recurrent EC have limited treatment options, though individualized therapy may emerge as another treatment consideration [7].

Originating from epithelial layers of the endometrial lining, EC is usually divided into two types based on histological characteristics, sex hormone receptor expression, and grade. Type 1 EC is the most common (approximately 80% of cases) and low-grade, also called estrogen-related endometrioid type. Type 2 EC is less common (approximately 20% of cases) and more aggressive, called estrogen-dependent non-endometrioid type [8,9]. In 2013, The Cancer Genome Atlas (TCGA) Research Network reported four new molecular subtypes using a comprehensive and integrated genomic profile of EC, including ultramutated DNA polymerase epsilon (POLE), hypermutated microsatellite instable (MSI), copy-number low (CNL), and copy-number high (CNH). These molecular subtypes have a significant impact on prognosis and treatment approach [10]. Based on this molecular subtype, the US Food and Drug Association (FDA) approved PD-1 inhibitors for treating mismatch repair deficiency (dMMR) or MSI and POLE molecular EC subtypes [11,12]. However, only 20–30% of EC patients with MSI molecular subtype respond to this drug, and it has less efficacy in microsatellite-stable (MSS) patients [13]. A recent study reported that dostarlimab, a PD-1 inhibitor, showed 43.5% and 14.1% objective response rates (ORRs) in MSI and MSS patients, respectively [14]. This suggests that in addition to MMR/MSI status, additional markers may emerge for evaluation for immune-checkpoint inhibitors (ICIs) response prediction. 

Our previous study using DNA methylation arrays and MethylCap-sequencing data from endometrial tissues found that the BHLHE22 promoter was highly methylated and, along with the CDO1 promoter methylation panel, was approved by the Taiwan FDA for early detection of EC using the conventional Papanicolaou smear sample [15,16]. BHLHE22, a basic helix loop helix transcription factor family member, acts as a transcriptional repressor and has a role in cell differentiation in neuron development [17,18]. To our knowledge, limited studies have investigated whether BHLHE22 expression is involved in cancer development, especially in EC. The role of BHLHE22 in EC tumorigenesis remains unclear. Nevertheless, HAND2, another BHLH family member, has a role in endometrial pathophysiology, which presents in the endometrial stromal cell and inhibits the ligand-dependent transcriptional activation function of estrogen receptor alpha (ER-*α*) and activates transcription of IL15 [19,20,21]. Furthermore, HAND2 expression is significantly lower in atypical hyperplasia and endometrioid carcinoma compared with normal endometrium [22]. BHLHE40, another BHLH family member, is highly expressed by TH1-like tumor-infiltrating CD4^+^ T cells, a population enriched in the microsatellite-instable subtype [23]. BHLHE40 expression is also inhibited by local PD-1 signaling and is important for tumor-infiltrating lymphocytes (TILs) reactivation after anti-PD-L1 inhibition [24]. Thus, we hypothesize that BHLHE22 may have a role in the immune microenvironment and thus confer clinical significance for the treatment of EC.

## 2. Results

### 2.1. BHLHE22 Is Downregulated in EC Tissues and Correlated with Poor Survival 

**Figure 1:**
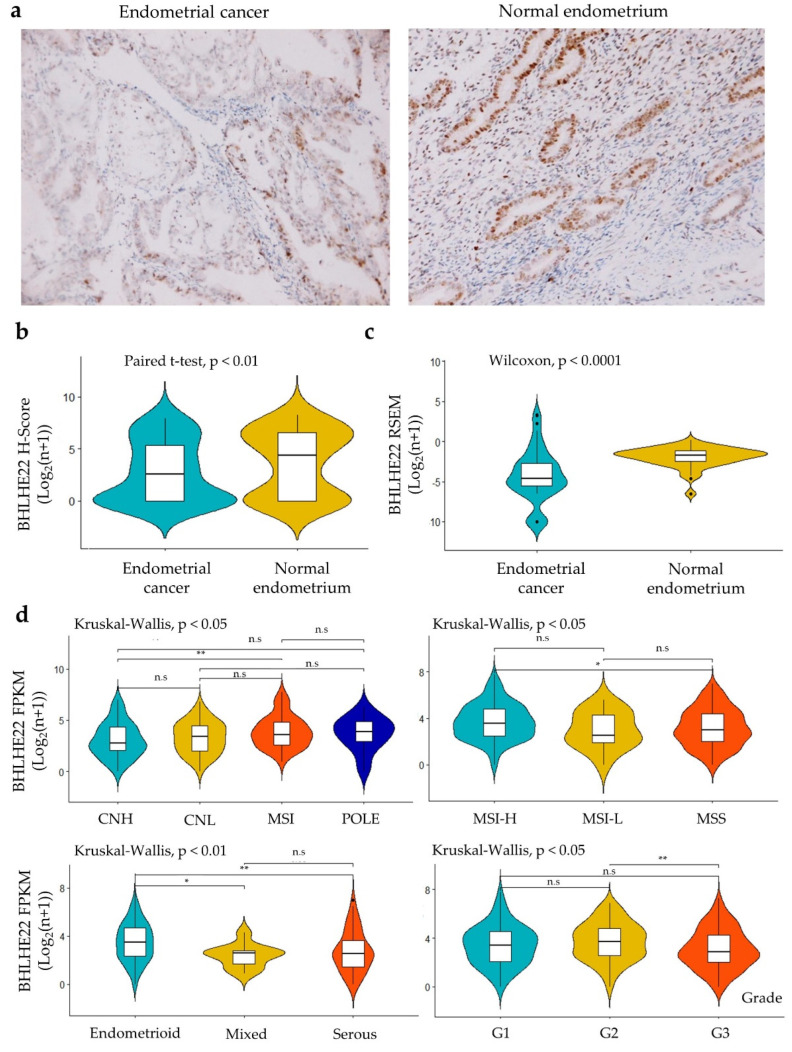
BHLHE22 expression was downregulated in endometrial cancer compared with normal endometrium tissues. (**a**). BHLHE22 protein expression levels in paired EC (left) and normal endometrium (right) were detected using IHC. Original magnification ×200. (**b**). BHLHE22 protein expression was quantified using the IHC H-score from our 54-pair EC tissue microarray. H-score was determined by adding the results of multiplication of the percentage of cells with staining intensity ordinal value (scored from 0 for “no signal” to 3 for “strong signal”) with 300 possible values. Paired sample *t* test is shown. (**c**). The BHLHE22 mRNA expression between EC (TCGA) and normal uterine (GTEx) is shown, the difference was tested using the Wilcoxon test. (**d**). BHLHE22 mRNA expression level is displayed based on patient characteristics using the UCEC TCGA database. Kruskal–Wallis test with Dunn’s multiple comparison test as a post hoc test is shown. TCGA, The Cancer Genome Atlas; GTEx, The Genotype-Tissue Expression; EC, Endometrial Cancer; POLE, DNA polymerase epsilon; MSI, microsatellite instability; CNH, copy-number high; CNL, copy-number low; MSI-H, microsatellite instability high; MSI-L, microsatellite instability low; MSS, microsatellite-stable. IHC, immunohistochemistry; RSEM, RNA-Seq by Expectation Maximization; FPKM, fragments per kilobase million; n.s., no-significant; *, *p* < 0.05; **, *p* < 0.001.

We analyzed BHLHE22 protein expression in 54 paired normal endometrium and EC tissues using immunohistochemistry (IHC) and found that BHLHE22 was significantly lower in EC tissue compared with normal (Figure 1a,b and Table S1). Consistent with our in-lab finding, the level of BHLHE22 was also significantly lower in EC compared with normal endometrium using RNA-Seq of the TCGA UCEC and GTEx databases (Figure 1c) and the human protein atlas database (Figure S1a,b). We found that MSI, POLE, endometrioid type, and low-grade were significantly associated with high BHLHE22 mRNA expression (Figure 1d), except staging and MLH1 silencing status (Figure S2a). We, therefore, tested the association between BHLHE22 expression and MSI-related DNA repair genes (MLH1, MSH2, MSH6, and PMS2). There was a significant positive correlation between BHLHE22 expression and MLH1 (*r* = 0.29; *p* < 0.05) (Figure S2b). EC endometrioid type had a significantly higher BHLHE22 expression than other histologic types (Figure 1d). BHLHE22 expression was negatively correlated with patient age (Figure S2c), suggesting that the downregulation of BHLHE22 might be related to aging in EC.

**Figure 2:**
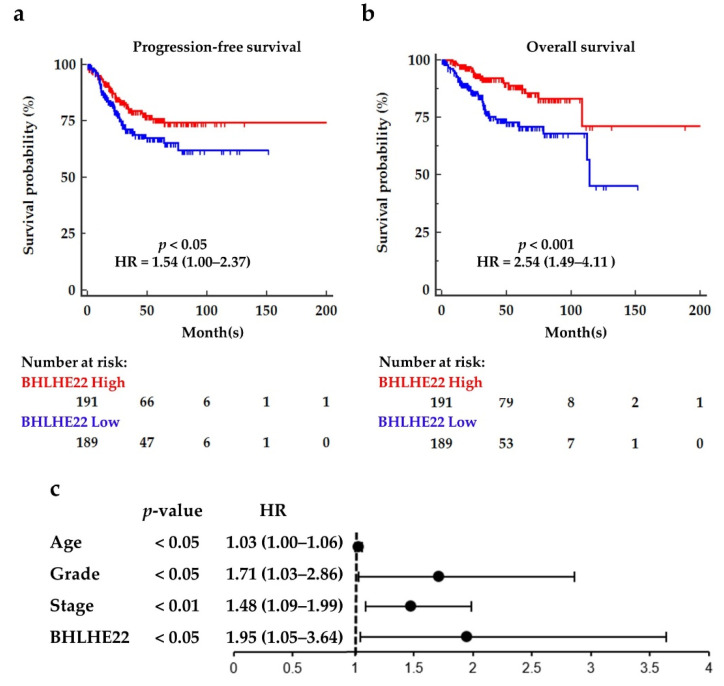
High BHLHE22 mRNA expression levels showed markedly favorable survival outcomes in EC. Kaplan–Meier analysis for progression-free survival (PFS, (**a**)), and overall survival (OS, (**b**)) of BHLHE22 mRNA expression in 380 EC patients were assessed using a Kaplan–Meier plotter. The BHLHE22^high^ patients (red line) revealed higher PFS and OS probability than BHLHE22^low^. Median BHLHE22 expression was used as the cut-off. Log-rank tests are shown. (**c**). BHLHE22 expression was an independent survival factor, after adjustment for stage, grade, and age. *p* value and HR (95%CI) are displayed. HR, hazard ratio.

Next, we assessed the survival outcomes of EC patients based on their BHLHE22 expression level using a Kaplan–Meier plotter. High expression of BHLHE22 conferred significant favorable progression-free survival (PFS) and overall survival (OS), with hazard ratios [HRs (95%CI)] of 1.54 (1.00, 2.37) and 2.54 (1.49, 4.11), respectively (Figure 2a). The median OS of low-BHLHE22 expression patients was 114 months but did not reach in the high-BHLHE22 expression patients. Five-year PFS rates were 77% and 69% for high and low BHLHE22 expression patients, respectively. Moreover, BHLHE22 expression was an independent factor in survival outcome in multivariate analysis adjusted for age, grade, and stage (Figure 2b). Altogether, BHLHE22 was downregulated and associated with endometrioid type, grade, and MSI in EC. High BHLHE22 expression conferred better survival. In addition, downregulation of BHLHE22 was observed in different tissues, including brain, lung, breast, cervix uteri, colon, and skin cancer using TCGA and GTEx databases (Figure S2d), implying a universal role of BHLHE22 in cancer biology.

### 2.2. BHLHE22 Expression Inhibits EC Proliferation and Migration

**Figure 3:**
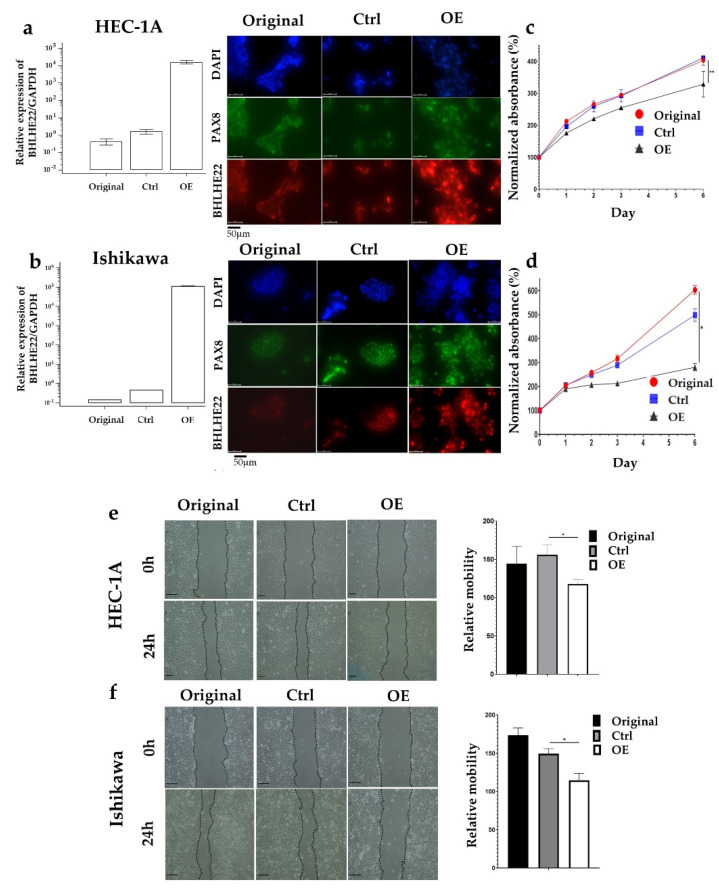
BHLHE22 inhibited the malignant phenotypes of EC cells. BHLHE22 was over-expressed in HEC1A (**a**) and Ishikawa (**b**) EC cell lines after transfection with pFUW-BHLHE22 plasmid. The expression of BHLHE22 was measured using quantitative RT-PCR (left) and immunofluorescence (right). Empty vector (ctrl) was set as 1. (**c**,**d**). Cell proliferation assay was significantly lower in BHLHE22 over-expressed cells compared with control using MTS assay. Ctrl was set as 100% at Day 0. (**e**,**f**). Relative mobility was significantly lower in over-expressed BHLHE22 cells compared with control using a wound-healing assay. Relative mobility was normalized to control. All results are shown as mean ± SD of triplicate experiments in each of two independent experiments. *p* values were measured by one-way ANOVA. OE, BHLHE22 Over Express; Ctrl, Control vector; h, hours; *, *p* < 0.05; **, *p* < 0.01.

To investigate the effects of BHLHE22 in EC, we over-expressed BHLHE22 in HEC1A and Ishikawa EC cell lines after transfection with pFUW-BHLHE22 plasmid. The mRNA and protein level of BHLHE22 was verified using quantitative RT-PCR and immunofluorescence staining (Figure 3a,b). We found a significant decrease in cell proliferation rate in BHLHE22 over-expressed EC cell lines compared with control and original cell lines (Figure 3c,d), which showed a 103.6% and 217.9% decrease in cell proliferation in HEC1A and Ishikawa cell lines, respectively. In addition, we also found a significantly lower relative mobility in BHLHE22 over-expressed EC cell lines compared with control and original cell lines using wound-healing assay (Figure 3e,f), which showed a 38.5% and 34.8% decrease in HEC1A and Ishikawa cell lines, respectively. Altogether, BHLHE22 was downregulated in EC, which lost prohibition of cell proliferation and migration, in turn, conferring poor survival.

### 2.3. BHLHE22 Is Associated with Immune Function in EC

**Figure 4:**
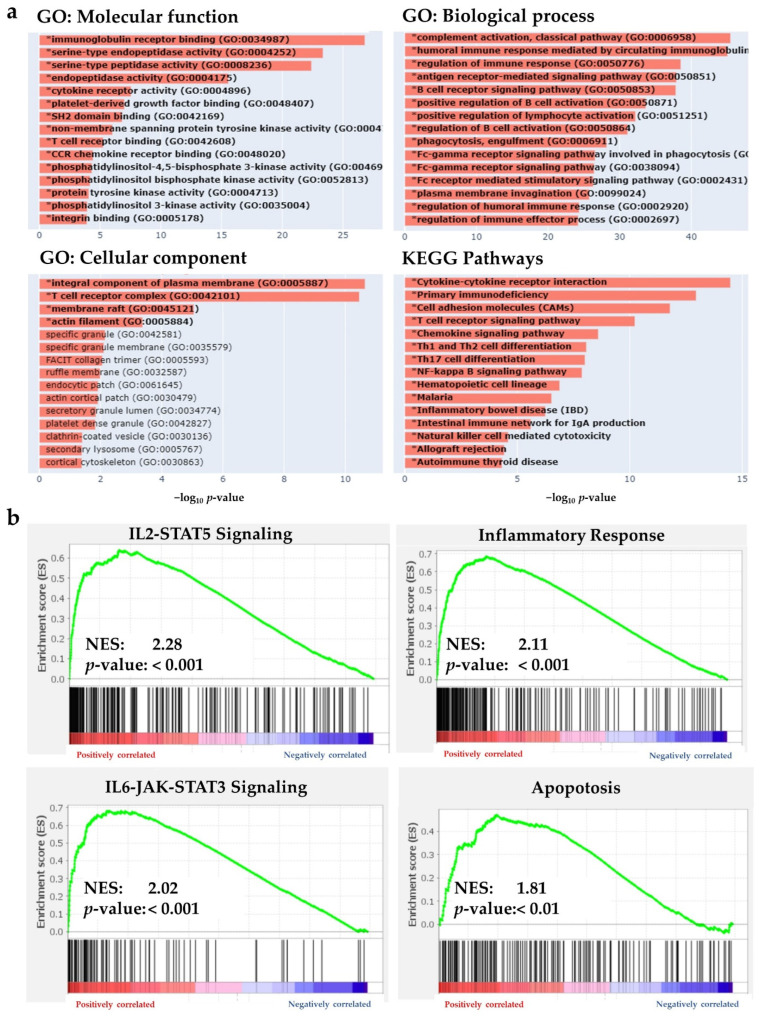
BHLHE22 expression enriched immune-related pathways in EC patients. (**a**). High BHLHE22 expression patients had markedly enriched immune-related pathways using gene ontology analysis using the TCGA UCEC dataset. (**b**). High BHLHE22 expression patients also significantly enriched in IL2-STAT5 signaling, inflammatory response, IL6-JAK-STAT3 signaling, and apoptosis by GSEA of cancer hallmark gene set signatures. GO, gene ontology; GSEA, gene set enrichment analysis; NES, Normalized Enrichment Score; KEGG, Kyoto Encyclopedia of Genes and Genomes.

Because BHLHE22 is a transcription factor, we investigated BHLHE22-associated genes (co-expressed and co-suppressed) using RNA-seq from the TCGA UCEC database. We found 1830 differentially expressed genes (DEGs) between high and low BHLHE22 expression by the median value, shown in the volcano plot (Figure S3a). The top 50 genes that positively or negatively correlated with BHLHE22 are shown in heatmaps (Figure S3b,c). To investigate the potential biological function of BHLHE22 in EC, gene set enrichment analysis (GSEA) [25] and gene ontology (GO) analysis of the DEGs of BHLHE22 were performed. We found that high expression of BHLHE22 in patients significantly increased immune-related biological function (Figure 4a), such as immunoglobulin receptor binding (false discovery rate (FDR) = 4.7 × 10^–25^) and cytokine receptor activity (FDR = 1.7 × 10^–6^) in molecular function, T cell receptor complex (FDR = 2.3 × 10^–9^) in a cellular component, complement activation (FDR = 6.3 × 10^–43^) and regulation of immune response (FDR = 1.8 × 10^–36^) in biological processes, and cytokine–cytokine receptor interaction (FDR = 6.5 × 10^–13^), T cell receptor signaling pathway (FDR = 2.8 × 10^–9^) and chemokine signaling pathway (FDR = 9.7 × 10^–8^) in the KEGG pathway. We also found that the top six significantly enriched gene sets related to immune response (FDR < 0.01) in the high BHLHE22 group, including IL2-STAT5 signaling (Normalized Enrichment Score, NES = 2.28), complement (NES = 2.28), allograft rejection (NES = 2.25), inflammatory response (NES = 2.11), IL6-JAK-STAT3 signaling (NES = 2.02), and apoptosis (NES = 1.82) by GSEA (Figure 4b).

**Figure 5:**
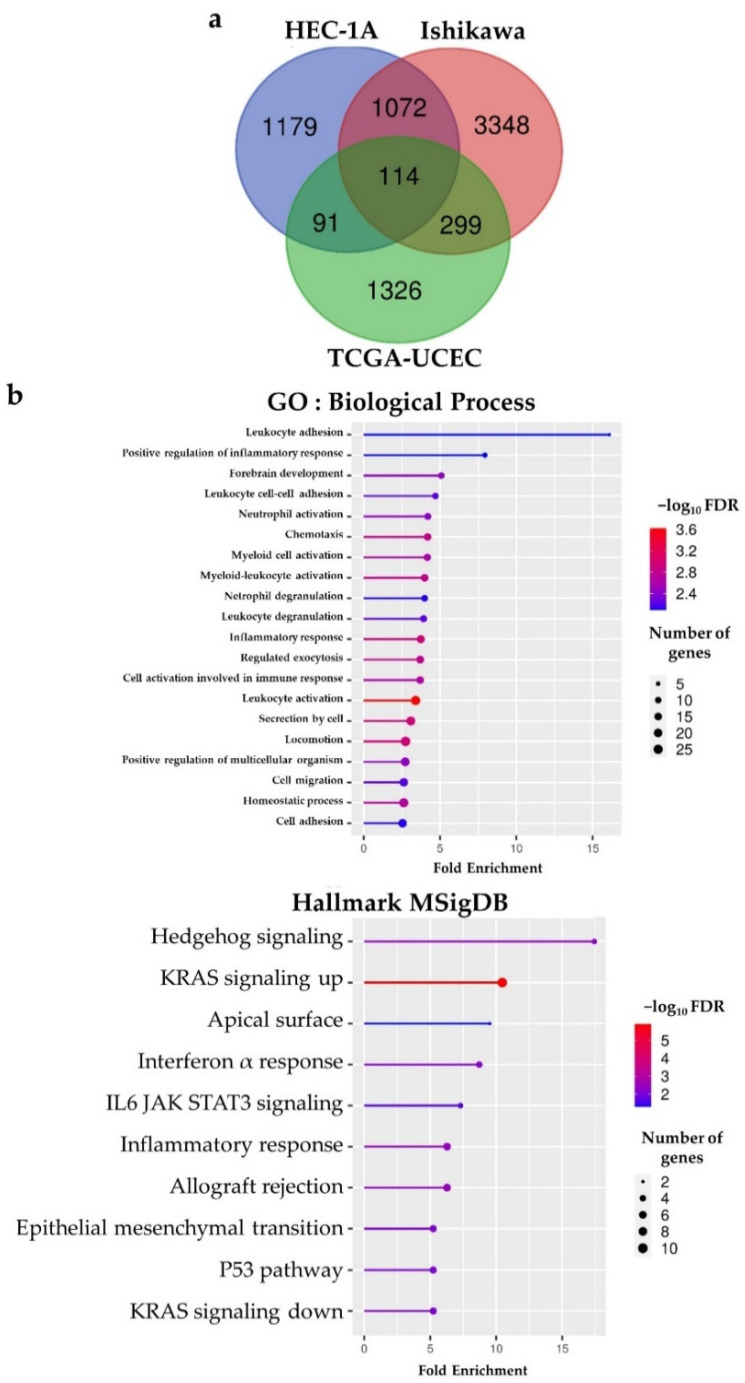
BHLHE22 expression in cell lines and patients confirmed enrichment of immune-related pathway in EC. (**a**). Venn diagram of differentially expressed genes (DEGs) in HEC1A and Ishikawa cell lines, and TCGA UCEC database based on the BHLHE22 expression showed 114 intersect genes among them. (**b**). Gene ontology and gene set enrichment analysis of the 114 DEGs confirmed enrichment of immune-related pathways of BHLHE22. MSigDB, The Molecular Signatures Database; UCEC, uterine corpus endometrial cancer; GO, gene ontology; FDR, false discovery rate.

We also performed RNA-seq analysis of BHLHE22 over-expressed cell lines, then selected genes that were differentially expressed with criterion |log_2_ (fold change)| ≥ 1. We found 4833 and 2357 DEGs in Ishikawa and HEC1A cell lines, respectively. When combined with DEGs from the TCGA database, we found 114 intersection genes after BHLHE22 expression (Figure 5a and Table S2). The ShinyGO tool [26] was used to analyze the gene set enrichment among 114 candidate genes, and BHLHE22 was significantly associated with leukocyte adhesion, regulation of inflammatory response, chemotaxis and myeloid cell activation in the biological process by GO, as well as interferon alpha response, IL6 JAK STAT3 signaling, and inflammatory response by hallmark gene set of MSigDB (Figure 5b). Cumulatively, these data suggest that BHLHE22 is involved in the EC immune regulatory network.

### 2.4. BHLHE22 Positively Correlated with Chemokines Expression and Proinflammatory Immune Cells in EC Microenvironment

**Figure 6:**
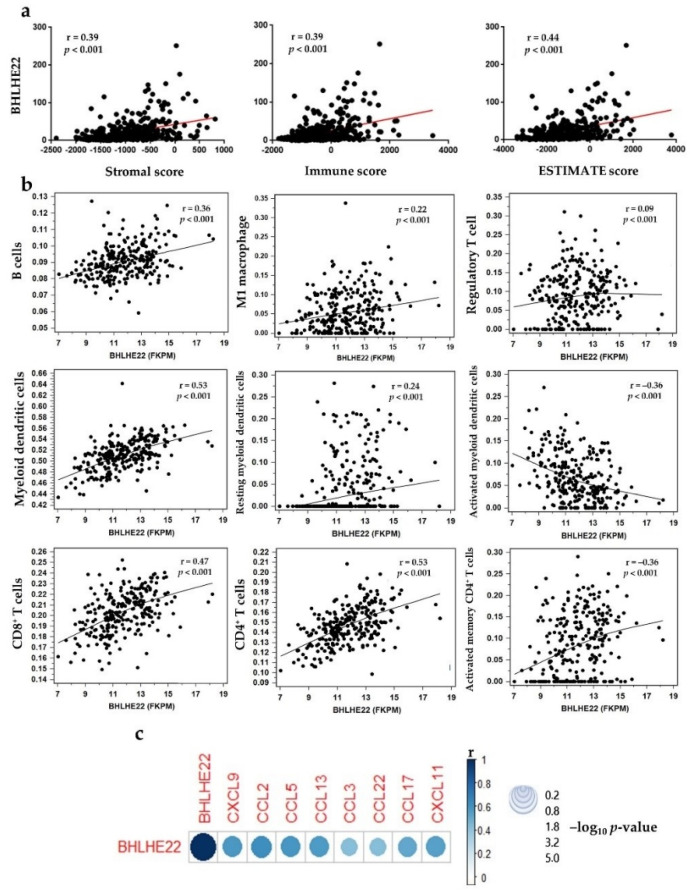
BHLHE22 expression level was associated with TIICs and correlated with chemokines’ expression in the EC microenvironment. (**a**). Stromal, immune, and ESTIMATE scores were positively correlated with BHLHE22 expression level in EC patients using the UCEC TCGA database. (**b**). BHLHE22 expression level correlated with inflammatory tumor-infiltrating immune cells in EC patients using the TIMER database. (**c**). The BHLHE22 expression level was strongly, positively correlated with the expression of chemokine genes in EC patients using the UCEC TCGA database. *p* and *r* values were measured using Pearson’s correlation. Circle size represents the –log_10_(*p* value) and color represents the *r* value.

Because BHLHE22 enriched immune-related genes in EC cell lines and patients, we analyzed whether BHLHE22 expression level was associated with leukocyte infiltration in EC. We computed the infiltration level of immune cells using the ESTIMATE algorithm [27] and discovered that the stromal, immune, and ESTIMATE scores were strongly and positively correlated with BHLHE22 expression in EC (Figure 6a), suggesting that BHLHE22 may be a regulator of host immune response, resulting in a hot immunogenic microenvironment. We further investigated tumor-infiltrating immune cells related to BHLHE22 expression in the UCEC TCGA database using TIMER and EPIC [28,29], which could predict and estimate the abundance value of tumor-infiltrating immune cells (TIICs) from the gene expression profile. BHLHE22 expression was significantly, positively correlated with immune cells infiltrate in the tumor microenvironment, including B cells, M1 macrophage, regulatory T cells, myeloid dendritic cells, resting myeloid dendritic cells, CD8^+^ T cells, CD4^+^ T cells, and activated memory CD4^+^ T cells, and negatively correlated with activated myeloid dendritic cells (Figure 6b and Figure S4a).

Because BHLHE22 was associated with inflammatory leukocyte infiltration, we further investigated the expression of chemokines and their correlation with BHLHE22 expression in the UCEC TCGA database. We found that expression of CXCL9, CCL2, CCL5, CCL13, CCL3, CCL22, CCL17, and CXCL11 was significantly and strongly positively correlated with BHLHE22 expression level (*r*^2^ > 0.5) (Figure 6c). Further, other immune-related genes that were positively correlated with BHLHE22 included CD274 (known as PD-1), CTLA4, CXCR5, FOXP3, ICOS, and LCK, with high correlation coefficients (*r*^2^ > 0.5) (Figure S4b). Altogether, these data suggest that BHLHE22 expression increased proinflammatory leukocyte infiltration by upregulating expression of chemokines, resulting in a hot tumor microenvironment.

## 3. Discussion

Herein, we show evidence supporting our hypothesis that BHLHE22 expression serves as a potential biomarker for ICI treatment prediction and favorable prognosis, and that is associated with microenvironment immune status. We found that BHLHE22 expression is associated with MSI. Previous evidence showed that MSI led to higher mutation rates and was considered a good biomarker for predicting ICI response in solid tumors, especially in EC [11,30]. However, a recent EC clinical trial reported that MSS patients also showed 14.1% objective response [14]. In addition, MSI and MSS patients share similar TIICs in EC [31]. Thus, BHLHE22 expression may be considered a novel biomarker to predict ICI response in EC, regardless of the MSI status.

BHLHE22 is a member of intronless genes, which are essential modulators of regulatory processes and have been proposed to be not only related to neuro-specific function but also to cancer, neuropathies, and developmental diseases [32]. Based on a neural development study, BHLHE22 is suggested as being involved in cell differentiation by forming a repressor complex with Prdm8 and regulated Cadherin-11 [17,18]. However, the investigation of BHLHE22 function in clinical outcomes of cancer has been limited. A recent breast cancer study reported that BHLHE22 is a signature gene, not only related to survival but closely associated with immunological responses [33]. Our present study highlighted that MSI EC is associated with BHLHE22 expression, which based on TCGA study, has a better survival compared to CNH [9,10]. Other BHLH family members are reported in cancer biology and clinical outcomes, especially brain cancers like ASCL1 in glioma and neuroblastoma, Olig2 in astrocytoma, and ATOH1 in medulloblastoma [34]. However, reports of BHLHE22 in EC clinical characteristics are rare. The present results reveal that BHLHE22 may be a potential, independent prognostic factor in EC. 

The role of BHLHE22 in EC remains largely unknown. We found significant roles of BHLHE22 in EC, including high BHLHE22 enriched immune-related pathways, such as IL2-STAT5 signaling, complement, allograft rejection, inflammatory response, IL6-JAK-STAT3 signaling. Furthermore, our study revealed a significant positive correlation between BHLHE22 expression and increased inflammatory TIILs, such as B cells, M1 macrophages, CD8^+^ T cells, CD4+ T cells, and myeloid dendritic cells in the EC microenvironment. Dendritic cell (DC) and CD8^+^ T cell infiltration is associated with favorable survival of EC [35]. We also found a strong positive correlation between BHLHE22 expression and chemokine genes that may attract macrophages, such as CCL2, CCL3, CCL5, CCL17, and CCL22 [36]. Chemokines that could attract CD8^+^ T cells also have a strong positive correlation with the expression of BHLHE22, such as CCL2, CCL17, CXCL9, and CXCL11. Previous studies have reported that increasing these chemokines is associated with increased CD8^+^ T cells in various cancers, including melanoma, ovarian, colorectal, and prostate cancers [37,38,39,40]. These chemokines are also reported to have beneficial functions in attaining a “hot” tumor microenvironment, potentially becoming therapeutic targets for future combinations with ICIs [41]. We also found a significant positive correlation between CD274 (PD-1) and CTLA4 as immune checkpoints with BHLHE22 expression. These findings improve the opportunity for targeting PD1/PDL-1 and CTLA4 in cancer biology, especially in EC, which already has a promising target in cancer treatment [42,43,44]. These results revealed the immune-modulatory role in EC, which may be a common phenomenon across other cancer types. 

## 4. Materials and Methods

### 4.1. Clinical Samples

To investigate the protein expression level of BHLHE22 in EC and normal tissues, we collected 107 EC and 57 normal endometrium tissues, including 54 paired normal endometrium and EC in the Taipei Medical University-Shuang Ho Hospital, New Taipei City. Ethical approval for the study was granted by the Institutional Review Board of Taipei Medical University-Shuang Ho Hospital, in conform to the Declaration of Helsinki. Informed consent was obtained from all subjects.

### 4.2. Cell Lines Culture Condition and Generation of Stable Cell Lines

HEC1A and Ishikawa were acquired from the Food Industry Research and Development Institute (FIRDI, Taipei, Taiwan) and the Japanese Collection of Research Bioresources Cell Bank (JCRB, Ibaraki city, Osaka, Japan). HEC1A cell lines were cultured in McCoy’s 5A (HyClone, Logan, UT, USA) with sodium pyruvate, supplemented with 10% fetal bovine serum (FBS) and 1% penicillin-streptomycin (Thermo Fisher Scientific, Waltham, MA, USA). Ishikawa cell lines were cultured in MEM (Welgene, Kyungsan, Korea) with 2 mM of glutamine, 1% non-essential amino acids, 10% FBS, and 1% penicillin-streptomycin (Thermo Fisher Scientific, Waltham, MA, USA). Cells were cultured in a humidified chamber with 5% CO_2_ at 37 °C.

BHLHE22 (NM_152414.5) was constructed by inserting a full-length cDNA product into a pFUW vector. One microgram of plasmid DNA was then transfected into Ishikawa and HEC1A cells with Lipofectamine 3000 (Thermo Fisher Scientific, Waltham, MA, USA) in Opti-MEM I reduced-serum medium (Thermo Fisher Scientific, Waltham, MA, USA) at 37  °C in a 5% CO_2_ atmosphere for 4–5 h, after which the medium was removed and replaced with fresh culture medium. Cells were selected by a Zeocin antibiotic (Thermo Fisher Scientific, Waltham, MA, USA) after transfection two days. Control cells were transfected with pFUW vector only. 

### 4.3. RNA Extraction, cDNA Synthesis, and Quantitative Real-Time PCR (qPCR)

Total RNA was isolated by Trizol reagent (Thermo Fisher Scientific, Waltham, MA, USA), counted using nanodrop, and then DNA was removed by DNAse (Thermo Fisher Scientific, Waltham, MA, USA). The Transcription First Strand cDNA Synthesis Kit (Roche, Basel, Swiss) was used to reverse-transcribed mRNA into cDNA for RT-PCR with Oligo (dT) as the primer. qPCR was performed by Roche SYBR Green Real-Time PCR System (Roche, Basel, Swiss). The glyceraldehyde-3-phosphate dehydrogenase (GAPDH) was used as the internal reference control. Relative quantification of mRNA expression was conducted using the comparative threshold cycles (Ct) method. All values were performed in triplicate and expressed as mean  ±  standard deviation (SD). The BHLHE22 primers used in this study are 5′-CAG CAG CAA GAA ATC CAA AGA GCA-3′ (forward) and 5′-CCA GCG CGT CGT TCA GGT C-3′ (reversed).

### 4.4. RNA Sequencing

Total RNA was quantified using a Nanodrop ND-1000 spectrophotometer (Thermo Fisher Scientific, Waltham, MA, USA), then submitted to the Health GeneTech Corp (Taoyuan, Taiwan) for next-generation sequencing. Libraries were prepared using Stranded RNA library was constructed by Swift RNA library Kit (Swift Biosciences, Cambridge, UK), according to the manufacturer’s protocols. Specifically, 5 μg of purified mRNA was reverse transcribed to cDNA. A tailing and ligation were performed to add adapter. Finally, a few cycles of PCR were performed to enrich the library. The quality and quantity of library were confirmed by gel electrophoresis, Qubit HS DNA assay, and qPCR measurement. Each library was paired-end sequenced (2 × 150 bp) on a Nova Seq 6000 platform (Illumina, San Diego, CA, USA). The RNA reads were then aligned to the human reference genome hg38. Relative gene expression was quantified as transcripts per million (TPM).

### 4.5. Immunofluorescence (IF)

Cells were fixed in 4% paraformaldehyde in PBS for 15 min at room temperature, washed twice with PBS, permeabilized in 0.1% Triton X in PBS for 10 min, and blocked with 5% BSA in PBS for one hour at room temperature. Cells were incubated with primary antibodies for one hour at room temperature, washed two times for 5 min with PBS, and incubated for one hour at room temperature with secondary antibodies diluted 1:400 in PBS, washed two times for 5 min with PBS. DAPI was added to the mounting medium. The following antibodies and dilution were used for immunofluorescence: BHLHE22 (1:200; HPA067500; S Merck KGaA, Darmstadt, Germany) and PAX8 (1:400; 10336-1-AP; Proteintech, Manchester, UK).

### 4.6. Cell Proliferation Assay

Cell proliferation was measured using MTS assay (Promega, Madison, WI, USA). Briefly, cells were seeded in 96-well plates at a density of 2000 cells/well. The culture medium was replaced by a fresh medium containing MTS reagent (90 µL medium plus 10 µL MTS reagent/well). After incubation at 37 °C for 1–4 hours, the absorption was measured at wavelengths of 490 nm. Experiments were performed with six replicates and measured on Days 1, 2, 3, and 6.

### 4.7. Wound Healing Assay

Cell migration was assessed using the scratch wound-healing assay. Cells were seeded into 6-well dishes until confluent and then wounded using a pipette tip. The migration area was photographed at 0 and 24 h (below the cell lines doubling time). Cell migration was quantified by measuring the migrated area using ImageJ. All experiments were performed in triplicate and expressed as mean ± SD.

### 4.8. Data Sources

We collected the transcriptome RNA-seq and clinical characteristics data of 373 UCEC patients from the TCGA database via https://www.cbioportal.org/ [10,45], accessed on 23 June 2021. Normal endometrium expression profile was downloaded from GTEX database through https://xenabrowser.net/ [46], accessed on 15 June 2021. Xena browser was also applied to analyze overall and relapse-free survival and validated using Kaplan-Meier Plotter web tool (https://kmplot.com/ [47], accessed on 22 Augustus 2021). We also collected BHLHE22 protein expression from the human protein atlas database (https://www.proteinatlas.org/, accessed on 27 March 2022) [48]. 

### 4.9. LinkedOmics Database and Gene Set Enrichment Analysis (GSEA)

We used linkedOmics web tool (http://www.linkedomics.org/) accessed on 2 July 2021, which includes TCGA UCEC database, to identify differential expression genes (DEG) related to high and low BHLHE22 expression levels based on their median value [49]. The volcano map, heat map, scatter map, and GO analysis of the co-expression of BHLHE22 were analyzed via the LinkedOmics function module using Pearson’s correlation coefficient. Furthermore, ShinyGO and GSEA tools were used to analyze 32,284 gene sets in the Molecular Signature Database (MSigDB) related to cancer phenotypes hallmark [25,26].

### 4.10. TIMER and EPIC Database Analysis

We used Tumor IMmune Estimation Resource (TIMER) to systematically analyze immune cells infiltrating associated with BHLHE22 expression such as in B cells, CD4^+^ T cells, CD8^+^ T cells, neutrophils, macrophages, and dendritic cells in UCEC TCGA database (https://cistrome.shinyapps.io/timer/, accessed on 13 June 2021). It could speculate the abundance value of tumor-infiltrating immune cells (TIICs) from the gene expression profile using the deconvolution method [28]. We validate it by using the Estimate the Proportion of Immune and Cancer (EPIC) tool to estimate the proportion of various immune cell types from bulk tumor gene expression data (http://epic.gfellerlab.org/, accessed on 10 July 2021) [29]. 

### 4.11. Statistical Analysis

The association between the expression of BHLHE22 and clinical features were evaluated by the logistic regression and Wilcoxon signed-rank test. The ANOVA test was used to identify the differences in proliferation rate and migration ability of BHLHE22 over-expressed EC cell lines. BHLHE22 expression data were analyzed using cBioportal R-package. Survival analysis was produced by the Xenabrowser, validated using Kaplan-Meier plotter web tools, and recalculated using MedCalc^®^ software (version 18.9.1, Ostend, Belgium). The correlation of gene expression was evaluated in the LinkedOmics and TIMER databases using Spearman’s correlation analysis and recalculated using R. Other data were assessed using Student’s *t*-test for two variables and one-way ANOVA for more than two variables. Data were presented as mean ± SD, and *p*-value < 0.05 was considered statistically significant.

## 5. Conclusions

BHLHE22 may regulate immune-related pathways and modulate the immune status of the EC microenvironment. Further investigation of the immune-modulatory role of BHLHE22 in EC is warranted.

## Data Availability

The data presented in this study are available from the corresponding author upon reasonable request.

## Supplementary Materials

The following supporting information can be downloaded at: https://www.mdpi.com/article/10.3390/ijms23137158/s1.

## References

[1] Zhang S., Gong T.T., Liu F.H., Jiang Y.T., Sun H., Ma X.X., Zhao Y.H., Wu Q.J. (2019). Global, Regional, and National Burden of Endometrial Cancer, 1990–2017: Results From the Global Burden of Disease Study, 2017. Front. Oncol..

[2] Siegel R.L., Miller K.D., Jemal A. (2015). Cancer statistics, 2015. CA Cancer J. Clin..

[3] Siegel R.L., Miller K.D., Fuchs H.E., Jemal A. (2021). Cancer Statistics, 2021. CA Cancer J. Clin..

[4] McAlpine J.N., Temkin S.M., Mackay H.J. (2016). Endometrial cancer: Not your grandmother’s cancer. Cancer.

[5] Temkin S.M., Kohn E.C., Penberthy L., Cronin K.A., Rubinsak L., Dickie L.A., Minasian L., Noone A.M. (2018). Hysterectomy-corrected rates of endometrial cancer among women younger than age 50 in the United States. Cancer Causes Control.

[6] Surveillance, Epidemiology, and End Results (SEER) Program. Cancer Stat Facts: Uterine Cancer. https://seer.cancer.gov/statfacts/html/corp.html.

[7] Connor E.V., Rose P.G. (2018). Management Strategies for Recurrent Endometrial Cancer. Expert Rev. Anticancer Ther..

[8] Morice P., Leary A., Creutzberg C., Abu-Rustum N., Darai E. (2016). Endometrial cancer. Lancet.

[9] Lu K.H., Broaddus R.R. (2020). Endometrial Cancer. N. Engl. J. Med..

[10] Levine D.A. (2013). The Cancer Genome Atlas Research Network. Integrated genomic characterization of endometrial carcinoma. Nature.

[11] Howitt B.E., Shukla S.A., Sholl L.M., Ritterhouse L.L., Watkins J.C., Rodig S., Stover E., Strickland K.C., D’Andrea A.D., Wu C.J. (2015). Association of Polymerase e-Mutated and Microsatellite-Instable Endometrial Cancers With Neoantigen Load, Number of Tumor-Infiltrating Lymphocytes, and Expression of PD-1 and PD-L1. JAMA Oncol..

[12] Le D.T., Uram J.N., Wang H., Bartlett B.R., Kemberling H., Eyring A.D., Skora A.D., Luber B.S., Azad N.S., Laheru D. (2015). PD-1 Blockade in Tumors with Mismatch-Repair Deficiency. N. Engl. J. Med..

[13] Ott P.A., Bang Y.J., Berton-Rigaud D., Elez E., Pishvaian M.J., Rugo H.S., Puzanov I., Mehnert J.M., Aung K.L., Lopez J. (2017). Safety and Antitumor Activity of Pembrolizumab in Advanced Programmed Death Ligand 1-Positive Endometrial Cancer: Results From the KEYNOTE-028 Study. J. Clin. Oncol..

[14] Oaknin A., Gilbert L., Tinker A.V., Brown J., Mathews C., Press J., Sabatier R., O’Malley D.M., Samouelian V., Boni V. (2022). Safety and antitumor activity of dostarlimab in patients with advanced or recurrent DNA mismatch repair deficient/microsatellite instability-high (dMMR/MSI-H) or proficient/stable (MMRp/MSS) endometrial cancer: Interim results from GARNET-a phase I, single-arm study. J. Immunother. Cancer.

[15] Huang R.L., Su P.H., Liao Y.P., Wu T.I., Hsu Y.T., Lin W.Y., Wang H.C., Weng Y.C., Ou Y.C., Huang T.H. (2017). Integrated Epigenomics Analysis Reveals a DNA Methylation Panel for Endometrial Cancer Detection Using Cervical Scrapings. Clin. Cancer Res..

[16] Liew P.L., Huang R.L., Wu T.I., Liao C.C., Chen C.W., Su P.H., Wang H.C., Weng Y.C., Lai H.C. (2019). Combined genetic mutations and DNA-methylated genes as biomarkers for endometrial cancer detection from cervical scrapings. Clin. Epigenetics.

[17] Xu Z.P., Dutra A., Stellrecht C.M., Wu C., Piatigorsky J., Saunders G.F. (2002). Functional and structural characterization of the human gene BHLHB5, encoding a basic helix-loop-helix transcription factor. Genomics.

[18] Ross S.E., McCord A.E., Jung C., Atan D., Mok S.I., Hemberg M., Kim T.K., Salogiannis J., Hu L., Cohen S. (2012). Bhlhb5 and Prdm8 form a repressor complex involved in neuronal circuit assembly. Neuron.

[19] Li Q., Kannan A., DeMayo F.J., Lydon J.P., Cooke P.S., Yamagishi H., Srivastava D., Bagchi M.K., Bagchi I.C. (2011). The antiproliferative action of progesterone in uterine epithelium is mediated by Hand2. Science.

[20] Fukuda T., Shirane A., Wada-Hiraike O., Oda K., Tanikawa M., Sakuabashi A., Hirano M., Fu H., Morita Y., Miyamoto Y. (2015). HAND2-mediated proteolysis negatively regulates the function of estrogen receptor α. Mol. Med. Rep..

[21] Murata H., Tanaka S., Tsuzuki-Nakao T., Kido T., Kakita-Kobayashi M., Kida N., Hisamatsu Y., Tsubokura H., Hashimoto Y., Kitada M. (2020). The transcription factor HAND2 up-regulates transcription of the IL15 gene in human endometrial stromal cells. J. Biol. Chem..

[22] Buell-Gutbrod R., Cavallo A., Lee N., Montag A., Gwin K. (2015). Heart and Neural Crest Derivatives Expressed Transcript 2 (HAND2): A novel biomarker for the identification of atypical hyperplasia and Type I endometrial carcinoma. Int. J. Gynecol. Pathol..

[23] Cook M.E., Jarjour N.N., Lin C.C., Edelson B.T. (2020). Transcription Factor Bhlhe40 in Immunity and Autoimmunity. Trends Immunol..

[24] Li C., Zhu B., Son Y.M., Wang Z., Jiang L., Xiang M., Ye Z., Beckermann K.E., Wu Y., Jenkins J.W. (2019). The Transcription Factor Bhlhe40 Programs Mitochondrial Regulation of Resident CD8(+) T Cell Fitness and Functionality. Immunity.

[25] Subramanian A., Tamayo P., Mootha V.K., Mukherjee S., Ebert B.L., Gillette M.A., Paulovich A., Pomeroy S.L., Golub T.R., Lander E.S. (2005). Gene set enrichment analysis: A knowledge-based approach for interpreting genome-wide expression profiles. Proc. Natl. Acad. Sci. USA.

[26] Ge S.X., Jung D., Yao R. (2020). ShinyGO: A graphical gene-set enrichment tool for animals and plants. Bioinformatics.

[27] Yoshihara K., Shahmoradgoli M., Martínez E., Vegesna R., Kim H., Torres-Garcia W., Treviño V., Shen H., Laird P.W., Levine D.A. (2013). Inferring tumour purity and stromal and immune cell admixture from expression data. Nat Commun.

[28] Li T., Fan J., Wang B., Traugh N., Chen Q., Liu J.S., Li B., Liu X.S. (2017). TIMER: A Web Server for Comprehensive Analysis of Tumor-Infiltrating Immune Cells. Cancer Res..

[29] Racle J., de Jonge K., Baumgaertner P., Speiser D.E., Gfeller D. (2017). Simultaneous enumeration of cancer and immune cell types from bulk tumor gene expression data. Elife.

[30] Le D.T., Durham J.N., Smith K.N., Wang H., Bartlett B.R., Aulakh L.K., Lu S., Kemberling H., Wilt C., Luber B.S. (2017). Mismatch repair deficiency predicts response of solid tumors to PD-1 blockade. Science.

[31] Song Y., Gu Y., Hu X., Wang M., He Q., Li Y. (2021). Endometrial Tumors with MSI-H and dMMR Share a Similar Tumor Immune Microenvironment. OncoTargets Ther..

[32] Aviña-Padilla K., Ramírez-Rafael J.A., Herrera-Oropeza G.E., Muley V.Y., Valdivia D.I., Díaz-Valenzuela E., García-García A., Varela-Echavarría A., Hernández-Rosales M. (2021). Evolutionary Perspective and Expression Analysis of Intronless Genes Highlight the Conservation of Their Regulatory Role. Front. Genet..

[33] Zhu J., Shen Y., Wang L., Qiao J., Zhao Y., Wang Q. (2022). A novel 12-gene prognostic signature in breast cancer based on the tumor microenvironment. Ann. Transl. Med..

[34] Dennis D.J., Han S., Schuurmans C. (2019). bHLH transcription factors in neural development, disease, and reprogramming. Brain Res..

[35] Li B.L., Wan X.P. (2020). Prognostic significance of immune landscape in tumour microenvironment of endometrial cancer. J. Cell Mol. Med..

[36] Ono S.J., Nakamura T., Miyazaki D., Ohbayashi M., Dawson M., Toda M. (2003). Chemokines: Roles in leukocyte development, trafficking, and effector function. J. Allergy Clin. Immunol..

[37] Galon J., Costes A., Sanchez-Cabo F., Kirilovsky A., Mlecnik B., Lagorce-Pagès C., Tosolini M., Camus M., Berger A., Wind P. (2006). Type, density, and location of immune cells within human colorectal tumors predict clinical outcome. Science.

[38] Wang W., Kryczek I., Dostál L., Lin H., Tan L., Zhao L., Lu F., Wei S., Maj T., Peng D. (2016). Effector T Cells Abrogate Stroma-Mediated Chemoresistance in Ovarian Cancer. Cell.

[39] Mollica Poeta V., Massara M., Capucetti A., Bonecchi R. (2019). Chemokines and Chemokine Receptors: New Targets for Cancer Immunotherapy. Front. Immunol..

[40] Mughees M., Kaushal J.B., Sharma G., Wajid S., Batra S.K., Siddiqui J.A. (2022). Chemokines and cytokines: Axis and allies in prostate cancer pathogenesis. Semin. Cancer Biol..

[41] Nagarsheth N., Wicha M.S., Zou W. (2017). Chemokines in the cancer microenvironment and their relevance in cancer immunotherapy. Nat. Rev. Immunol..

[42] Sharma P., Allison J.P. (2015). Immune checkpoint targeting in cancer therapy: Toward combination strategies with curative potential. Cell.

[43] Grywalska E., Sobstyl M., Putowski L., Roliński J. (2019). Current Possibilities of Gynecologic Cancer Treatment with the Use of Immune Checkpoint Inhibitors. Int. J. Mol. Sci..

[44] Whelan S., Ophir E., Kotturi M.F., Levy O., Ganguly S., Leung L., Vaknin I., Kumar S., Dassa L., Hansen K. (2019). PVRIG and PVRL2 Are Induced in Cancer and Inhibit CD8(+) T-cell Function. Cancer Immunol. Res..

[45] Gao J., Aksoy B.A., Dogrusoz U., Dresdner G., Gross B., Sumer S.O., Sun Y., Jacobsen A., Sinha R., Larsson E. (2013). Integrative analysis of complex cancer genomics and clinical profiles using the cBioPortal. Sci. Signal..

[46] Goldman M.J., Craft B., Hastie M., Repečka K., McDade F., Kamath A., Banerjee A., Luo Y., Rogers D., Brooks A.N. (2020). Visualizing and interpreting cancer genomics data via the Xena platform. Nat. Biotechnol..

[47] Nagy Á., Munkácsy G., Győrffy B. (2021). Pancancer survival analysis of cancer hallmark genes. Sci. Rep..

[48] Sjöstedt E., Zhong W., Fagerberg L., Karlsson M., Mitsios N., Adori C., Oksvold P., Edfors F., Limiszewska A., Hikmet F. (2020). An atlas of the protein-coding genes in the human, pig, and mouse brain. Science.

[49] Vasaikar S.V., Straub P., Wang J., Zhang B. (2018). LinkedOmics: Analyzing multi-omics data within and across 32 cancer types. Nucleic Acids Res..

