# Supplementary material for: BHLHE22 Expression Is Associated with a Proinflammatory Immune Microenvironment and Confers a Favorable Prognosis in Endometrial Cancer
Source: Int J Mol Sci. 2022 Jun 28;23(13):7158. doi: 10.3390/ijms23137158 (PMC9266305; doi:10.3390/ijms23137158)
Supplement: Supplementary file 1 [file ijms-23-07158-s001.zip › ijms-1764541-supplementary.pdf]

Supplemental Informations

BHLHE22 Expression is Associated with a Proinflammatory Immune Microenvironment and Confers a Favorable Prognosis in Endometrial Cancer

Darmawi, Lin-Yu Chen, Po-Hsuan Su, Phui-Ly Liew, Hui-Chen Wang, Yu-Chun Weng, Rui-Lan Huang and Hung-Cheng Lai

Supplemental tables

Table S1. Patient clinical characteristics

**Clinical characteristics**		**n**	**Mean ± SEM H-score of BHLHE22**	***p*-value**
Age (years)	55 (34-92)	107	42.1 ± 6.25	**<0.05 (r = 0.21)** *
Stage	I	74	33.38 ± 57.37	**n.s** **
	II	12	54.17 ± 77.37	
	III	14	42.14 ± 64.92	
	IV	1	80	
	N/A	6	119.17 ± 86.39	
Histologic type	Endometrioid	95	35.26 ± 60.85	**<0.01** **
	Mixed	6	116.67 ± 44.57	
	Serous	6	75.83 ± 89.58	
Grade	1	54	28.98 ± 55.87	**<0.05** **
	2	33	41.06 ± 62.87	
	3	20	79.25 ± 77.41	
Limphvascular invasion (LVI)	Negative	62	39.19 ± 60.86	**n.s** **
	Positive	43	48.14 ± 70.97	
	N/A	2	2.5 ± 3.54	
FIGO	IA	62	35.81 ± 58.88	**<0.05** **
	IB	15	66 ± 84.03	
	II	14	57.5 ± 77.11	
	IIIA	3	11.67 ± 10.41	
	IIIC	11	34.09 ± 58.17	
	IVB	1	80	
	N/A	1	0	

*** Pearson test; ** One-way ANOVA.**

Table S2. Differential expressed gene list among HEC1A, Ishikawa and TCGA UCEC related to BHLHE22 expression

	Gene names			
Upregulated	BHLHE22	KLHDC7B	CSTA	CNTN4
	CXCR5	TMEM158	HCST	CPZ
	HOXC10	MILR1	INS-IGF2	GPSM3
	SPIB	CXCL8	CLEC4A	ATP2A3
	SELL	HOXC4	ALPK2	LTB
	NRG1	SLC12A3	F5	LRRK2
	LGALS2	TMEM37	IGFL2	ARHGAP9
	PRKCB	RFTN1	LINC00494	SLC7A7
	CD27	F2R	CD68	CDH2
	MARCO	SYNE1	SLC38A5	TMED6
	IL2RG	RSAD2	MSLN	PTGES
	CD52	PTGER4	GLI1	DPYSL2
	CLRN3	PAPPA	CSF2RA	ZNF506
	ITGB2	KCNAB2	ANKRD44	CDK6
	EBI3	ETS1	ALDH1A3	NT5E
	FGD2	NXPH4	FBLN2	DOCK11
	ISL2	MMP25	ALOX5	SEMA3D
	LAMP3	HLA-DRB1	ST6GAL2	RAB39B
	CCL3L3	VNN1	ADGRD1	COPZ2
	ACSM5	SLC9A2	SUCNR1	KRT17
	ISM1	LOXL4	ATP2C2	NELL2
	UTS2	CTSS	ADAM8	DAB2
	KRT23	IFI44L	SDS	HERC5
	LINC00882	RASSF5		
Downreguated	MAST1	SERPINA3		
	ZYG11A	TNNI1		
	LINC00336	RPPH1		
	SERPINA4	SFTPD		
	GSC	SERPINB5		
	LEMD1	SLC34A3		
	PALM3	DLX5		
	GPC2	CAMK2B		
	ABCC6P2	NMU		
	BTNL9	KLK8		

Supplemental figures

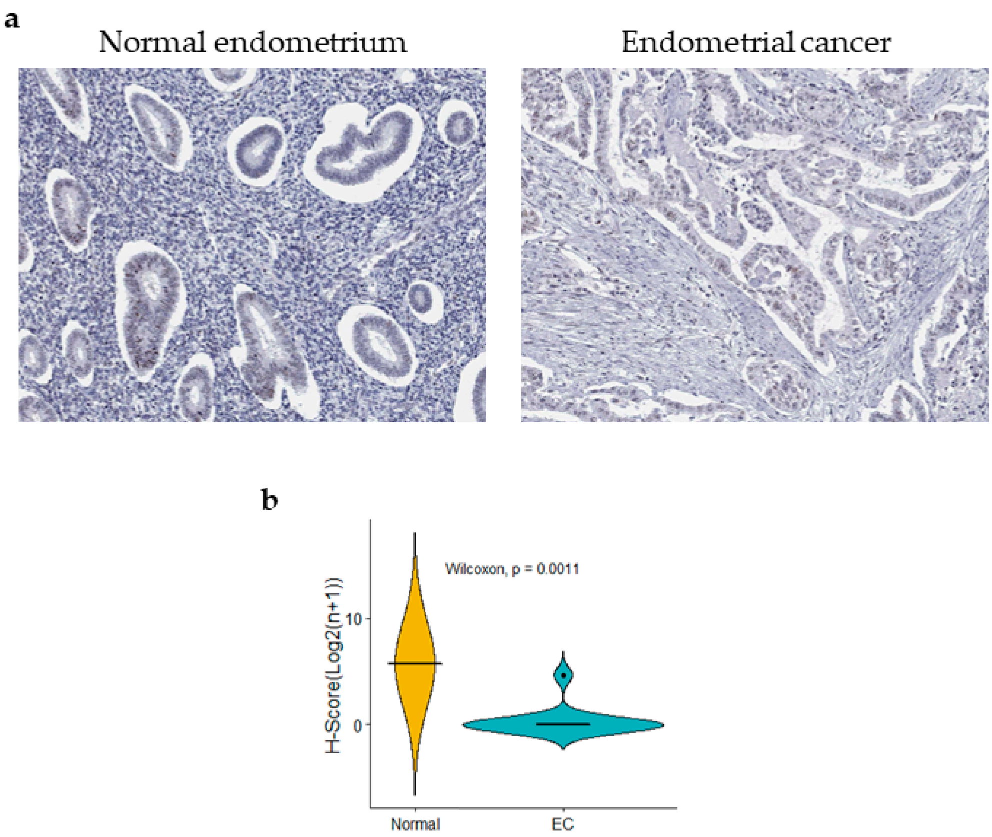

Figure S1. BHLHE22 expression was downregulated in endometrial cancer compare to normal endometrium tissues.

BHLHE22 protein level in normal endometrium and endometrial cancer from IHC of the Human Protein Atlas (HPA) database (a) and quantitatively bu H-score (b). The statistical analysis was tested by Wilcoxon-test. IHC, Immunohistochemistry.

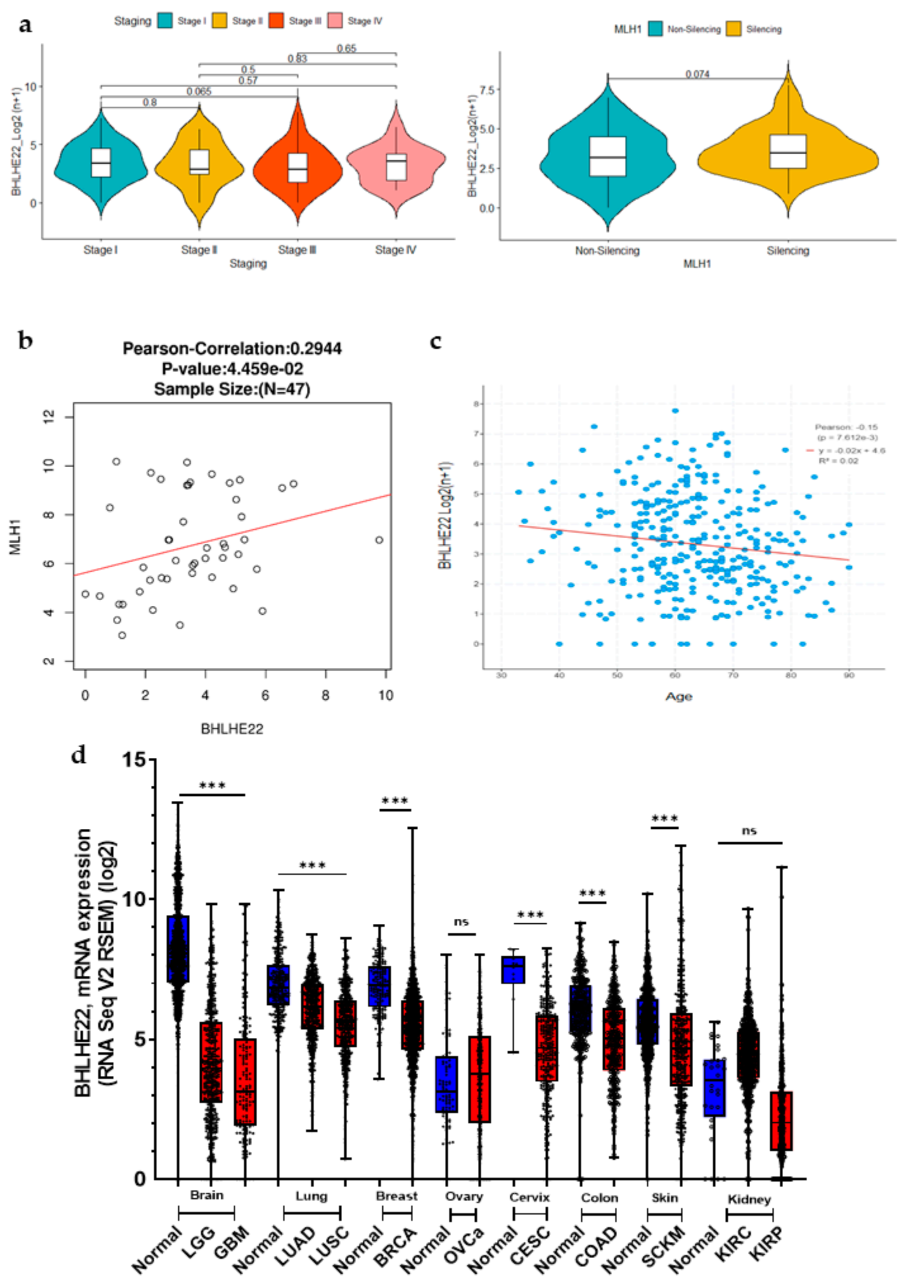

Figure S2. BHLHE22 was associated with clinical characteristics and generally downregulated in tumor compare to normal.

a. Association between clinical characteristic and BHLHE22 expression were assessed in EC patients using UCEC TCGA database. b. Correlation of BHLHE22 expression and MMR related gene, MLH1, were examined in EC patients. c. BHLHE22 expression were negatively correlated with age in EC patients. Pearson-correlation coefficient were shown. d. BHLHE22 mRNA expression in other cancers compared with their normal tissues were tested using Xena browser. TCGA, The Cancer Genome Atlas; LGG, Low Grade Glioma; GBM, Glioblastoma Multiform; LUAD, Lung Adenocarcinoma; LUSC, Lung Squamous Cell Carcinoma; BRCA, Breast Invasive

Carcinoma; OVCa, Ovarian Cancer; CESC, Cervical Squamous Cell Carcinoma and Endocervical Adenocarcinoma; UCEC, Uterine Corpus Endometrial Carcinoma; COAD, Colon adenocarcinoma; SCKM, Skin Cutaneous Melanoma; KIRC, Kidney Renal Clear Cell Carcinoma; KIRP, Kidney Renal Papillary Cell Carcinoma; TPM, transcript per millions; EC, Endometrial Cancer; POLE, DNA polymerase epsilon; MSI, microsatellite instability; MSS, microsatellite stable; *, p<0.05; **, p<0.01; ***, p<0.001; ****, p<0.0001.

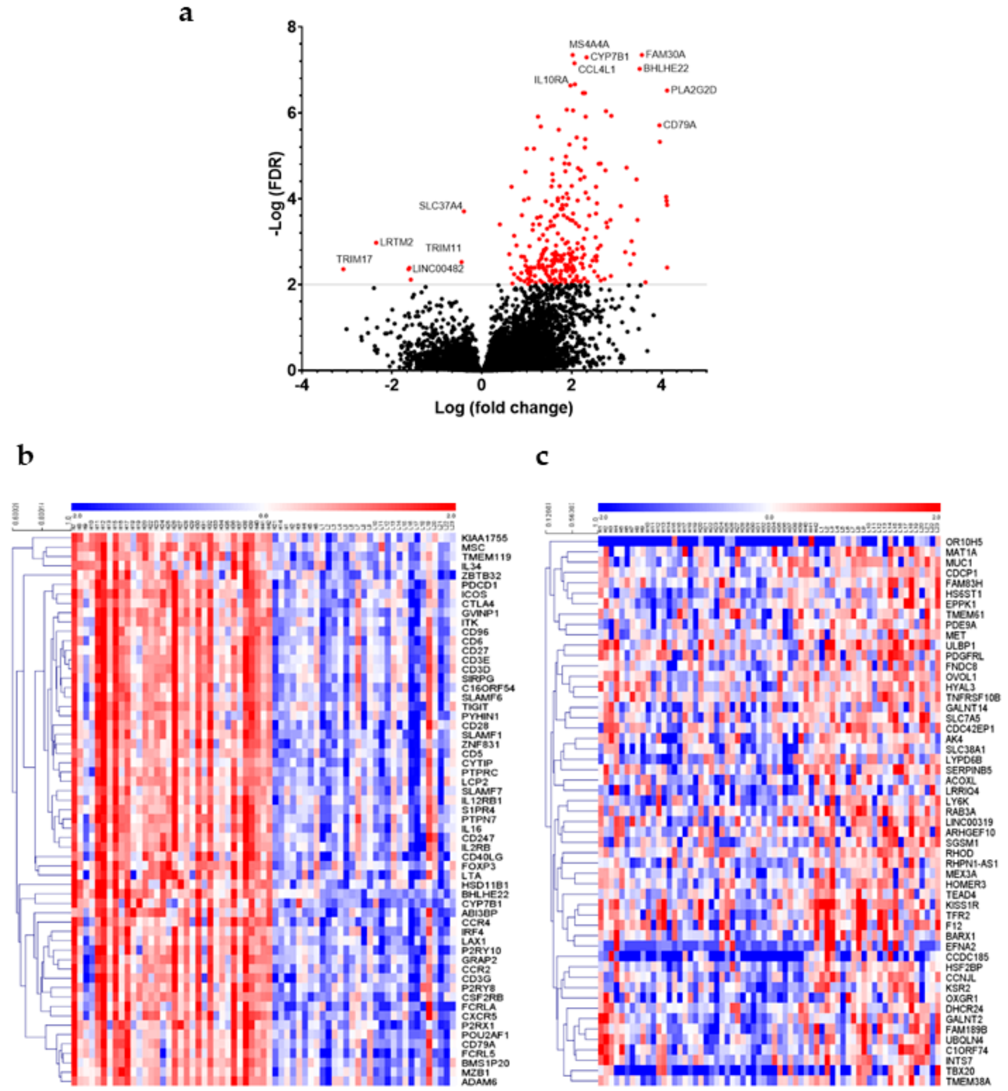

Figure S3. Genes were affected by the high BHLHE22 mRNA expression in EC patients.
a. Differential expressed genes (DEGs) of EC patients using TCGA UCEC dataset based on the BHLHE22high expression level were shown in volcano plot. Heatmaps showed top 50 DEGs, which were positively (b) or negatively (c) correlated with BHLHE22 expression level in EC patients, respectively.

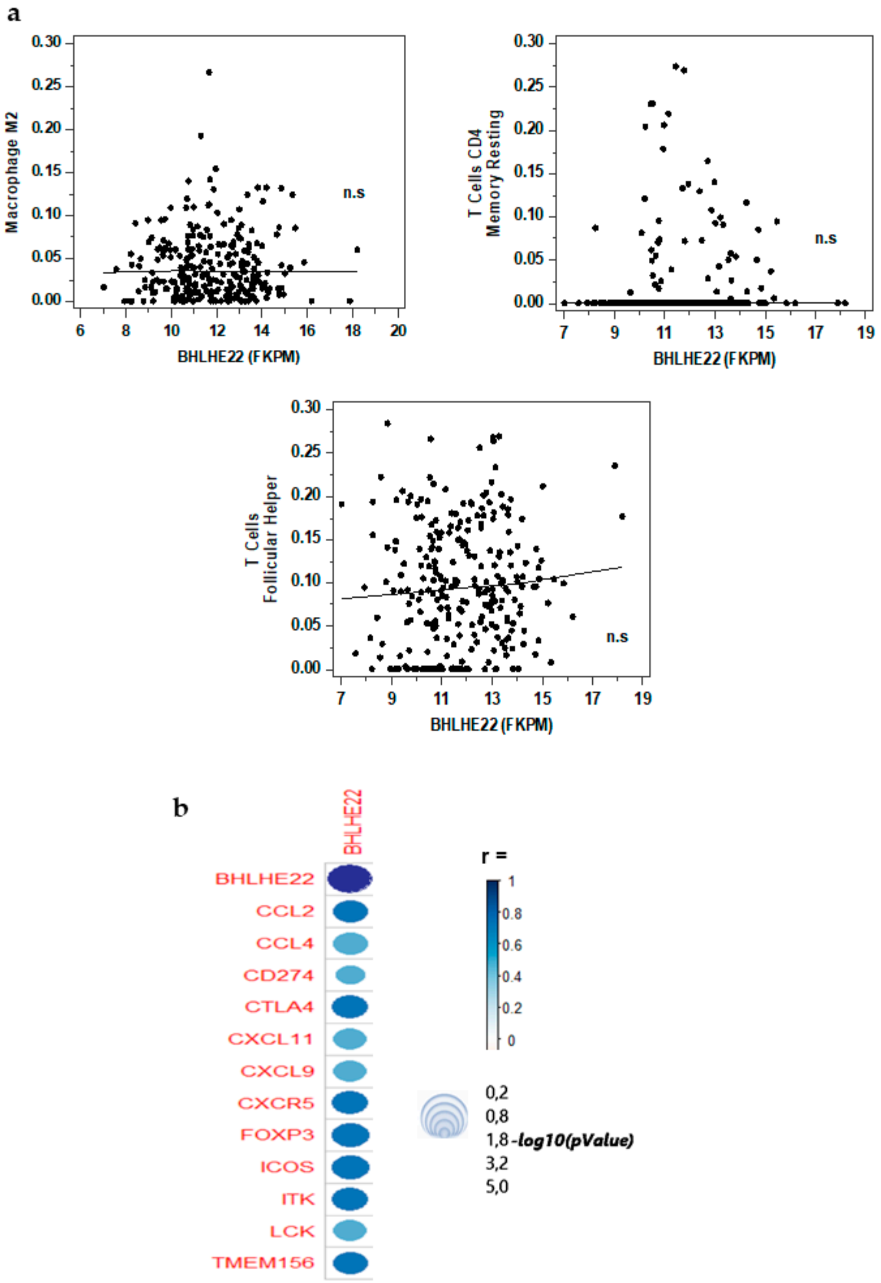

Figure S4. The association between BHLHE22 expression and other immune cells in EC patients were as-sessed by TIMER algorism.

a. BHLHE22 expression level was unsignificantly correlated with macrophage M2, T cells memory resting and T cell follicular helper. Statistical anaysis with Pearson correlation test were shown. b. BHLHE22 expression had a strong positive correlation with immune-related genes expression in EC patients using UCEC TCGA database. Circle size represented the –log10(p-value) and color represented r value.
